# Selection and validation of reference genes for quantitative gene expression normalization in *Taxus* spp.

**DOI:** 10.1038/s41598-020-79213-1

**Published:** 2020-12-17

**Authors:** Kaikai Zhang, Wei Fan, Duanfen Chen, Luyuan Jiang, Yunfeng Li, Zhiwang Yao, Yanfang Yang, Deyou Qiu

**Affiliations:** 1 State Key Laboratory of Tree Genetics and Breeding, Key Laboratory of Tree Breeding and Cultivation of State Forestry Administration, The Research Institute of Forestry, Chinese Academy of Forestry, Beijing, 100091 China; 2 College of Horticulture, Agricultural University of Hebei, Baoding, 071001 China; 3 State Key Laboratory of Tree Genetics and Breeding, Chinese Academy of Forestry, Beijing, 100091 China; 4 College of Agriculture, South China Agricultural University, Guangzhou, 510642 China

**Keywords:** Molecular biology, Plant sciences

## Abstract

Quantitative real-time PCR (qRT-PCR) is commonly used to measure gene expression to further explore gene function, while suitable reference genes must be stably expressed under different experimental conditions to obtain accurate and reproducible data for relative quantification. Taxol or paclitaxel is an important anticancer compound mainly identified in *Taxus* spp. The molecular mechanism of the regulation of taxol biosynthesis is current research goal. However, in the case of *Taxus* spp., few reports were published on screening suitable reference genes as internal controls for qRT-PCR. Here, eight reference genes were selected as candidate reference genes for further study. Common statistical algorithms geNorm, NormFinder, BestKeeper, ΔCt, and RefFinder were used to analyze the data from samples collected from a cell line of *Taxus* × *media* under various experimental conditions and from tissues of *Taxus chinensis* var. *mairei*. The expression patterns of *TcMYC* under salicylic acid treatment differed significantly, with the best and worst reference genes in the cell line. This study screened out suitable reference genes (*GAPDH1* and *SAND*) under different treatments and tissues for the accurate and reliable normalization of the qRT-PCR expression data of *Taxus* spp. At the same time, this study will aid future research on taxol biosynthesis-related genes expression in *Taxus* spp., and can also be directly used to other related species.

## Introduction

Yew (*Taxus* spp.) is a medicinal tree distributed in many regions of the world. The antitumor drug paclitaxel (taxol) can be extracted from *Taxus* spp. Many researchers have reported that taxanes, including taxol, baccatin III, and 10-deacetylbaccatin III, can be extracted from the needles, stems, roots, and bark of yew trees^1–3^. Moreover, studies have shown that the presence of inductive treatments, such as coronatine (COR), methyl jasmonate (MeJA), salicylic acid (SA), and ethylene (ETH) can greatly enhance taxol content in *Taxus* spp.^4–7^. Furthermore, previous research indicates that abscisic acid (ABA) plays a critical role in ozone-induced taxol production by *T. chinensis* suspension cell-cultures^8^.

The molecular mechanism of the regulation of taxol biosynthesis is our research objective. Thus, we have previously reported on some of the genes encoding enzymes involved in taxol synthesis and transcription factors regulating such synthesis^9–11^. To investigate the expression of these taxol biosynthesis-related genes in *Taxus* spp., the qRT-PCR method was used commonly owing to its cost effectiveness, sensitivity, reproducibility, and simplicity. Although RNA-seq can yield information on gene expression, qRT-PCR has been usually used to investigate the gene expression pattern with relatively small number and to evaluate gene expression results of RNA-seq. In general, the two most common methods for qRT-PCR are absolute and relative quantification^12^. Absolute quantification is usually used to determine gene copy number, and relative quantification is commonly used to detect the transcripts level changes of target genes under different experimental conditions^13^. However, to obtain reproducible and reliable results, some experimental conditions must be satisfied in relative quantification qRT-PCR, such as gene specific primers, high-quality RNA without genomic DNA, and suitable reference genes, which have serious implications for the normalization of data^14^.

An ideal reference gene should have relatively stable expression across different developmental stages, distinct cell types, and various experimental conditions^15^. Therefore, it is necessary that stable reference genes are evaluated for each species under analysis; in this way, they can be assumed applicable to any experimental conditions in relative quantification qRT-PCR method^16–18^. However, the expression of many common reference genes changes under different conditions of time and space^15^. It is unlikely that a suitable universal reference gene will be adequate for different experiments. For instance, the expression of *18S rRNA* was stable in rice but has different results in papaya^19,20^. *Actin* was expressed stable in the study of tomato virus infection but could not be a reliable reference gene in cucumber under salinity stress or in papaya under numerous experimental condition^20–22^. Therefore, it is necessary to select and optimize reference genes for qRT-PCR according to the experimental material and treatments to improve the accuracy of qRT-PCR analysis and interpretation^14,23,24^.

Although there are many studies related to the function of genes encoding key enzymes involved in taxol biosynthesis, at present, there are only a few reference genes that are used in taxane-biosynthesis-related gene expression; for example, *GAPDH* (encoding Glyceraldehyde-3-phosphate dehydrogenase), *TBC41* (3,5-epimerase-4-reductase), and *18S rRNA* (18S ribosomal RNA)^25–27^. *Actin*, one of the most stable reference genes, was screened and evaluated across different tissues and abiotic stress conditions in many plants^28–32^, and was also used as the reference gene to investigate the expression pattern of genes involving in *Taxus* spp.^28^. Whether these reference genes are stable and suitable to different experimental conditions, such as MeJA, COR, and ABA, has not been evaluated. With unstable reference genes, it is difficult to get accurate results and reveal the mechanism of taxol synthesis. However, proper evaluation for stability of stable reference genes for qRT-PCR analysis in *Taxus* spp. is still lacking.

The analysis of taxol biosynthesis-related gene expression in tissues or under various inducing treatments can provide important clues to elucidate gene functions involved in taxol biosynthesis, which would be helpful in improving taxol content. Therefore, in this study, a set of eight candidate genes were evaluated under different inductive conditions and in various tissues of *Taxus* spp., aiming to provide stable reference genes that can help to achieve in accurate, relative quantification qRT-PCR analysis of gene expression to understand taxol-biosynthesis-related gene expression patterns and functions.

## Results

### Primers performance for amplifying the candidate reference genes

**Figure 1:**
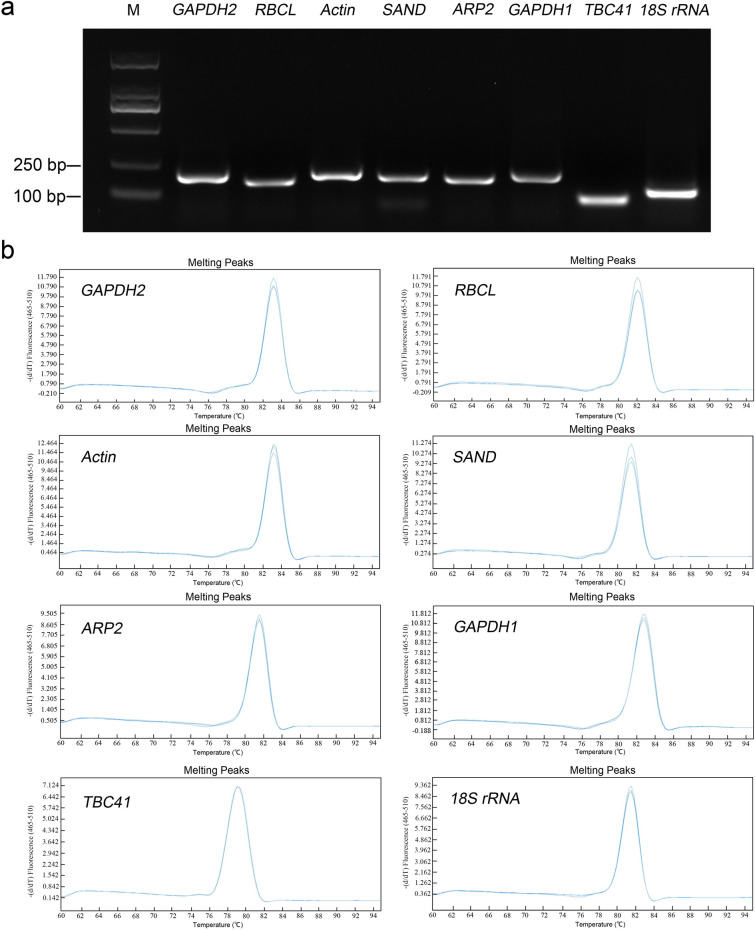
Amplification specificity of primers using qRT-PCR. (**a**) Cropped agarose gel of eight candidate reference genes PCR products, and full-length gel is presented in Supplementary Fig. S1. (**b**) Uniformity of melting curves of amplified product for each candidate reference gene. The (**a**) was generated by ChemiDoc Touch Gel Imaging System (version 1708370; https://www.bio-rad.com/), the (**b**) was generated by LightCycler480 software (version 1.2.0.169; https://lifescience.roche.com/en_cn.html).

By agarose gel electrophoresis, the PCR products of eight candidate reference genes were detected, and each obtain a single-specific expected size band (Fig. 1a). Consistently, a single peak was identified in the qRT-PCR melting curve analysis with each tested gene primer pairs (Fig. 1b). These results indicated that the test gene primer pairs had high specificity (Fig. 1a,b). Each primer pair amplification efficiency values varied between 92.5% and 103.9% with *R*^2^ ≥ 0.98 (Table 1). Thus, all the candidate genes primers used in this study were specific and effective.

**Table 1:** Primer and amplification efficiency (E) of candidate reference genes obtained by qRT-PCR.

Gene name	Annotation	Primer (5′–3′)	Length (bp)	Efficiency (%)	*R*^2^	Referehnces
*SAND*	Sand protein	F: TGGTGGATATTCTGAGGCTACA R: TCTGCGAGTGGAAGACCTAC	208	99.7	0.9882	–
*ARP2*	Actin-related protein	F: AACTACACGCTTCCAGATG R: TCCTCCGCTCAGAACAAT	192	93.7	0.9982	–
*RBCL*	Ribulose-1,5-bisphospate carboxylase/oxygenase	F: TTCGTTGGCTCATTATTGTC R: TTCTCGTTCTCCTTCAAGTT	187	103.9	0.9975	–
*GAPDH1*	Glyceraldehyde-3-phosphate dehydrogenase	F: TCAGCAGATGCACCGATGT R: GACCTCCACGCCAATCCTT	226	98.9	0.9985	–
*Actin*	Actin	F: AAGAGAAGCTTGCTTATGTAGC R: TCTGATATCCACATCACACTTC	200	92.5	0.9952	^28^
*18S rRNA*	18S ribosomal RNA	F: TCTGGTCCTGTTCCGTTGGC R: TGCTTTCGCAGTGGTTGTCTT	100	102.0	0.9998	^26^
*GAPDH2*	Glyceraldehyde-3-phosphate dehydrogenase	F: TTCCCTGGGGTGAGGTTGGT R: GCCAAAGGAGCCAGGCAGTT	229	96.4	0.9989	^25^
*TBC41*	3,5-epimerase-4-reductase	F: CAAGAAGAAAGAGTCAGCAAATGG R: GGAACGACATGACATTATGAATAGC	91	95.4	0.9859	^27^
*TcMYC*	MYC transcription factor	F: AAAAGAGGGAGAAAGCCTGC R: CACAGCCCTGAGTGCATAGA	111	90.8	0.9963	^9^

### Expression profile of the candidate reference genes

**Figure 2:**
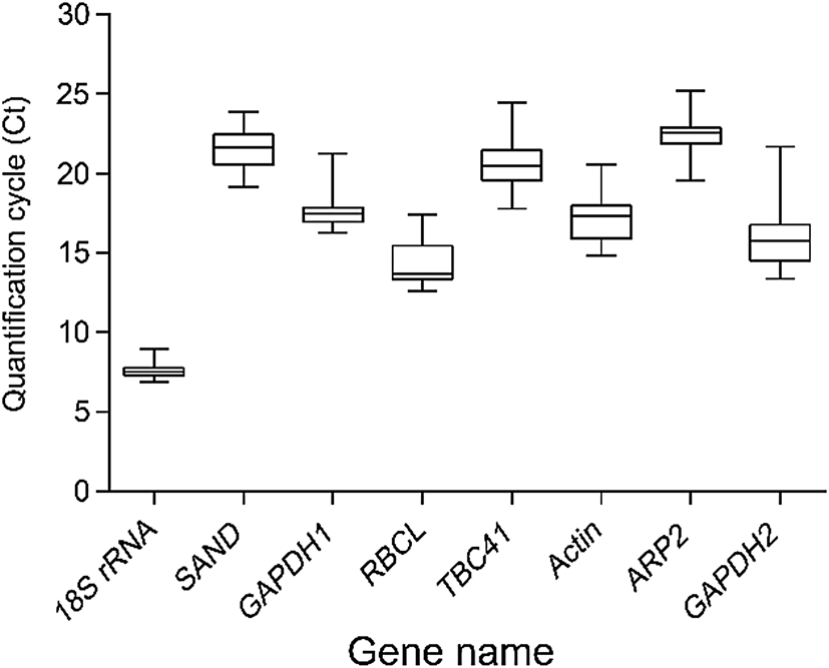
Ct values of eight candidate reference genes in all samples from various treatments and tissues. Boxes indicate the 25th and 75th percentiles in all samples; lines across the boxes represent the median; and the whisker caps represent the maximum and minimum values. The figure was generated by GraphPad Prism 9 (version 9.0.0; https://www.graphpad.com/).

Firstly, the distribution of cycle threshold (Ct) values of the eight candidate reference genes in all treatment and tissues samples was analyzed (Fig. 2). As shown on Fig. 2, the Ct values of candidate genes from each sample showed a high variation, which indicated that the expression of the highest was *18S rRNA* (6.92–8.98), while the lowest was *ARP2* (19.55–25.22).

According to mean value, standard deviation (SD), and coefficient of variation (CV) of Ct values, among the eight reference genes, *GAPDH2* (CV = 12.72%) showed the lowest stability, while *SAND* (CV = 4.90%) and *ARP2* (CV = 4.67%) showed relatively higher stability.

### Analysis of expression stability candidate reference genes

**Figure 3:**
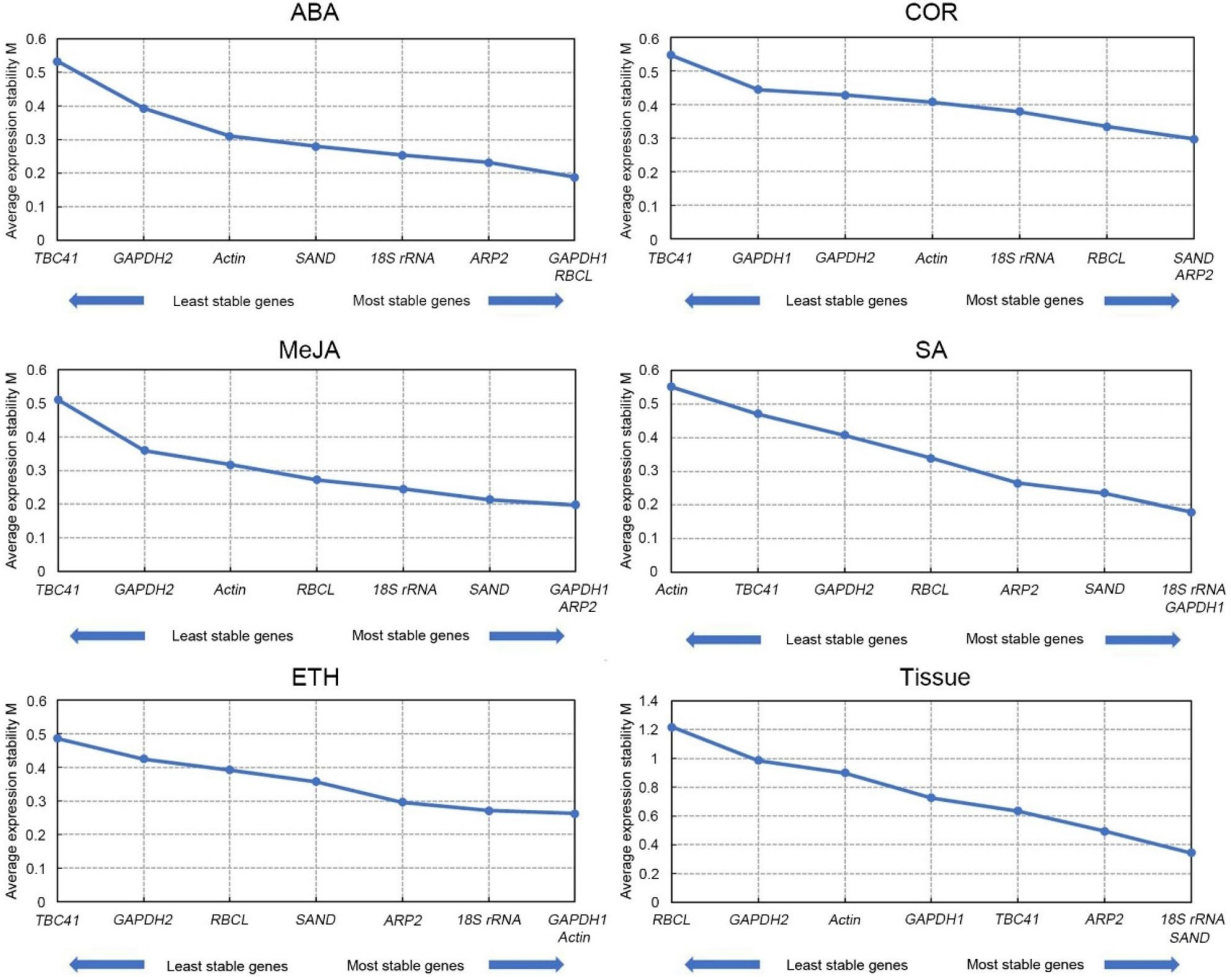
Gene expression stability and ranking of eight reference genes under different treatments and tissues based on geNorm. Expression stability value (M) for each reference gene was obtained and graphed. ABA, abscisic acid; COR, coronatine; MeJA, methyl jasmonate; SA, salicylic acid; ETH, ethylene. The figures were generated by geNorm (version 3.4; http://medgen.ugent.be/~jvdesomp/genorm/).

In this study, geNorm analysis indicated that the two most stable genes after the treatments of elicitors were as follows: in ABA treatment, *RCBL* (M = 0.189) and *GAPDH1* (M = 0.189); in COR treatment, *ARP2* (M = 0.298) and *SAND* (M = 0.298); in MeJA treatment, *GAPDH1* and *ARP2* (M = 0.198); similarly, *18S rRNA* and *GAPDH1* were the most stable genes inducing with SA (M = 0.180); while *GAPDH1* and *Actin* were the most stable genes after ETH treating (M = 0.262). On the other hand, the reference genes, *18S rRNA* and *SAND* were the most stable (M = 0.346) across all tissues studied (Fig. 3, Supplementary Table S1).

NormFinder analysis showed that *GAPDH1* (SV = 0.025) and *RCBL* (SV = 0.096) were the most stable under ABA treatment, thus confirming the results obtained by geNorm analysis. However, the results of these two programs were not all the same. Under COR treatment, the most suitable genes were *18S rRNA* (SV = 0.146) and *ARP2* (SV = 0.173); the most stable genes under MeJA and SA treatments were *GAPDH1* (SV = 0.097; SV = 0.117) and *SAND* (SV = 0.130; SV = 0.081), while under ETH treatment, *Actin* (SV = 0.129) and *18S rRNA* (SV = 0.139) were the most stable. As for the tissues studied, the expression of *18S rRNA* (SV = 0.052) and *SAND* (SV = 0.120) were the most stable in all of them, as was the case when geNorm was used for the analysis. Moreover, *TBC41* was the most unstable under ABA, COR, and ETH treatments, while *Actin* and *RBCL* was the most unstable under SA treatment and in the different tissues, respectively (Supplementary Table S1).

The SD results from BestKeeper analysis showed that *ARP2* was the most stable reference genes inducing by ABA and *RBCL* gene under COR and ETH treatments, while *18S rRNA* was the most suitable reference gene under MeJA and SA treatments, and in the different tissues sampled (Supplementary Table S1).

Here, ΔCt analysis showed that *RBCL*, *18S rRNA*, *GAPDH1*, and *Actin*, were the most stabilized genes under ABA, COR, MeJA, and ETH treatments, respectively, while *SAND* was the most stable reference gene under SA treatment and across tissues (Supplementary Table S1).

Finally, the stability of the eight candidate reference genes was compared based on the RefFinder tool which integrates the outputs of geNorm, NormFinder, BestKeeper, and the comparative Ct methods. According RefFinder analysis, *SAND* ranked as the most stabilized and *TBC41* as the least stabilized gene among all samples (Table 2). In addition, the results indicated that *RBCL*/*GAPDH1*, *18S rRNA*/*ARP2*, *GAPDH1*/*ARP2*, *GAPDH1*/*SAND*, and *Actin*/*GAPDH1*, were the most stable reference gene combinations under ABA, COR, MeJA, SA, and ETH treatments, respectively, while the combination *18S rRNA*/*SAND*/*ARP2* was most suitable in the different tissues.

**Table 2:** Stability and ranking of eight candidate reference genes analyzed by RefFinder.

Condition	*RBCL*	*GAPDH1*	*18S rRNA*	*ARP2*	*SAND*	*Actin*	*GAPDH2*	*TBC41*
ABA	1.565 (1)	1.682 (2)	2.913 (4)	2.632 (3)	5.000 (5)	6.000 (6)	7.000 (7)	8.000 (8)
COR	3.482 (4)	5.180 (6)	1.682 (1)	1.861 (2)	2.913 (3)	5.233 (7)	4.924 (5)	8.000 (8)
MeJA	4.401 (5)	1.316 (1)	2.991 (4)	2.000 (2)	2.913 (3)	6.481 (6)	6.481 (7)	8.000 (8)
SA	5.000 (5)	1.682 (1)	2.000 (3)	3.464 (4)	1.732 (2)	7.445 (8)	6.447 (6)	7.000 (7)
ETH	4.141 (5)	2.590 (2)	2.632 (3)	4.427 (6)	3.976 (4)	1.316 (1)	6.481 (7)	8.000 (8)
Tissue	8.000 (8)	6.435 (7)	1.189 (1)	3.224 (3)	1.414 (2)	5.692 (6)	4.120 (4)	5.180 (5)
All	5.000 (6)	2.000 (2)	3.834 (4)	2.280 (3)	1.682 (1)	4.120 (5)	7.238 (7)	7.737 (8)

The number within parentheses represents the ranking of the reference gene.

ABA, abscisic acid; COR, coronatine; MeJA, methyl jasmonate; SA, salicylic acid; ETH, ethylene; *SAND*, sand protein; *ARP2*, actin-related protein; *RBCL*, ribulose-1,5-bisphospate carboxylase/oxygenase; *GAPDH1/2*, glyceraldehyde-3-phosphate dehydrogenase; *18S rRNA*, 18S ribosomal RNA; *TBC41*, 3,5-epimerase-4-reductase.

### Optimal number of reference genes under different experimental conditions

**Figure 4:**
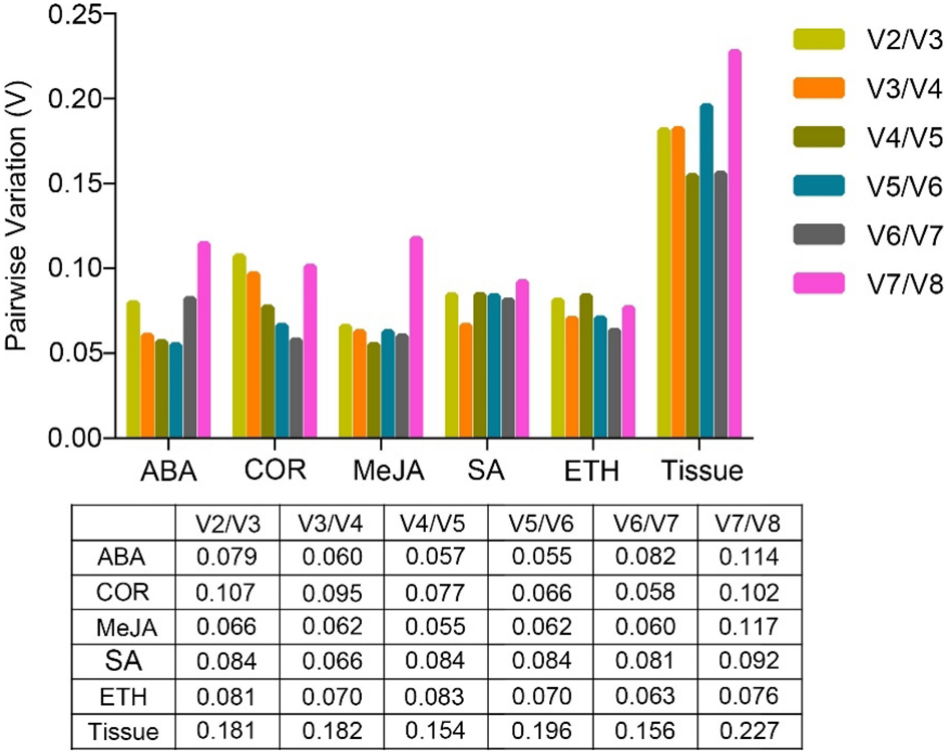
Optimal number of reference genes in different samples from various treatments and tissues using geNorm. Pairwise variation (V) was calculated to obtain the minimum number of reference genes required for normalization under each experimental condition. ABA, abscisic acid; COR, coronatine; MeJA, methyl jasmonate; SA, salicylic acid; ETH, ethylene. The figure was generated by GraphPad Prism 9 (version 9.0.0; https://www.graphpad.com/).

The optimum number of reference genes to suit specific experimental conditions can be provided by the geNorm software. When the V_n_/V_n+1_ ratio is less than 0.15, it indicates that the optimum number is n^33^. In this study, all values of the V_n_/V_n+1_ ratio under ABA, MeJA, COR, SA, and ETH treatments were lower than 0.15, indicating that two reference genes were suitable. Conversely, all values of the V_n_/V_n+1_ ratio in different tissues were greater than 0.15, with the lowest value calculated at 0.154 (V_4_/V_5_), thus suggesting that the three stable reference genes were suitable (Fig. 4).

### *TcMYC* expression pattern under SA treatment

**Figure 5:**
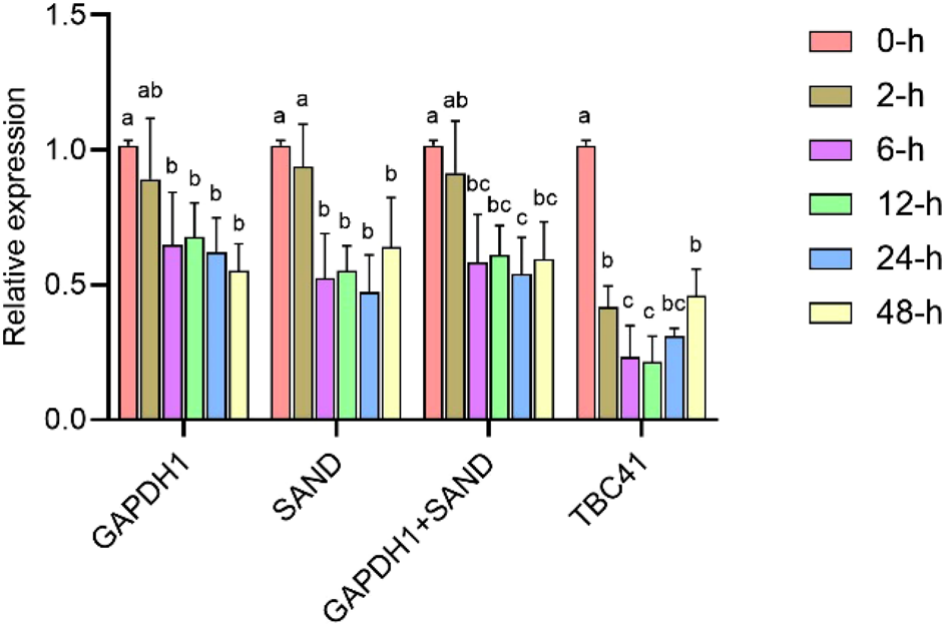
Relative quantification of *TcMYC* expression in *T.* × *media* cells induce by salicylic acid with two stable reference genes, *GAPDH1* and *SAND*, separately or in combination, and the unstable reference genes *TBC41*. The figure was generated by GraphPad Prism 9 (version 9.0.0; https://www.graphpad.com/).

The *TcMYC g*ene (accession number KC878013) was selected to examine its expression level under SA treatment with the two sets of the best and worst reference genes. As Fig. 5 shows, the expression pattern of *TcMYC* did not significantly change under SA treatment for 2 h with the use of the combination of *GAPDH1* and *SAND* as reference genes*.* Moreover, after induction by SA treatment for 6–48 h, there was no significant difference in the transcripts of *TcMYC*, which decreased and remained at a relatively stable level, about 0.5-fold that of the control (0 h), with *GAPDH1*/*SAND* used as reference genes. Although the results seemed very similar, the expression pattern of *TcMYC* was different with *GAPDH1* or *SAND* alone used as the unique reference gene, relative to when both *GAPDH1* and *SAND* were used as reference genes. For example, the transcripts of *TcMYC* were significantly fewer after induction by SA treatment for 12 h when compared with 2 h, with *GAPDH1*/*SAND* used as reference genes. However, there was no significant difference in the transcripts of *TcMYC* for 2–24 h with *GAPDH1* or *SAND* as reference gene. Furthermore, the transcript level of *TcMYC* was more inaccurate with the worst reference gene, namely, *TBC41*. With both *GAPDH1* and *SAND* used as reference genes, the transcripts of *TcMYC* decreased significantly and remained at a relatively stable level, about 0.5-fold that of the control (0 h) after induction by SA treatment for 6–48 h. The quantification of the expression level of *TcMYC* with *TBC41*, at all these sampling-time points of SA treatment changed clearly at 2, 6 and 12 h; at these timings, the transcripts of *TcMYC* at 2 h of SA treatment was clearly lower than that of the control (0 h), and they also were clearly lower at 6 and 12 h than that of 48 h.

## Discussion

Many reference genes have been used to investigate gene expression in a number of plants, including *Actin*^31,32^, *GAPDH*^25^, *Ubiquitin*^34^, and *18S rRNA*^26^. There is not one single stable and suitable gene for all experimental conditions, such as different tissues and development periods, as well as stress treatments^17,35,36^. Previous studies reported the selection and evaluation work of stable and suitable reference genes in plants is very important^15,29,35,37,38^, however, reports about the evaluation of stable reference genes in *Taxus* spp. are scarce. In this study, the transcripts of eight candidate reference genes changed greatly under corresponding experimental conditions and in different tissues; furthermore, stable reference genes and their combinations differed by the algorithms. These variations among the five tools are an expected result due to the differences in statistical algorithms used in each program^39^. In order to overcome their individual limitations and ensure a more reliable evaluation of the data, it was suggested that the suitable reference genes were chosen according to the results of RefFinder, which takes into consideration the rankings of the other four software methods (geNorm, NormFinder, BestKeeper, ΔCt method). Moreover, it was also suggested to consider the reference gene or their combinations stability obtained by at least three programs^39,40^.

Currently, the consensus is that multiple reference genes are more accurate and reliable than any single gene for revealing the gene expression level^41,42^. The suitable reference genes number can be confirmed with the pairwise variation (V value) analyzed by geNorm. By default, when V_n_/V_n+1_ < 0.15, the suitable reference genes number is n. In this study, all V_2_/V_3_ values were less than 0.15 under ABA, COR, MeJA, SA, and ETH treatments, indicating that using two reference genes were suitable under these experimental conditions. The V_n_/V_n+1_ values of various tissues were larger than 0.15, indicating that a new reference gene should be added in examining gene expression patterns in tissues of *Taxus* spp., besides the top two reference genes given by geNorm. The reason for this observation might be the large differences among tissue types; similar results were reported for maritime pine (*Pinus pinaster* Aiton)^14^. Three stable reference genes, *18S rRNA*, *SAND*, and *ARP2*, were suggested for use in future investigations of gene expression in different tissues in *Taxus* spp.

The traditional reference gene, *18S rRNA*, was considered one of the least stable reference genes among common reference genes analyzed in many plants^37,43,44^. For example, *18S rRNA* was evaluated and performed poorly in 29 samples different developmental processes and stress treatments (cold, heat, and salt) as well as organs in *Lilium*^28^. Moreover, in strawberry (*Fragaria* × *ananassa*), it was not recommended for normalization under specific experimental conditions due to low stability^45^. In contrast, the performance of *18S rRNA* was not the worst in this study, where it ranked first in the evaluation for RefFinder’s stability under COR treatment and across tissues, as per RefFinder. Although *TBC41* performed more stable in *T.* × *media* cell cultures elicited by MeJA and M-β-cyclodextrin, separately or in combination^46^, it was less stable in this study under ABA, COR, MeJA and ETH treatments. These results clearly showed that it is necessary to evaluate reference genes before starting relative quantification, according to the experimental conditions.

Some novel reference genes were selected in the current study with better performance compared to the common housekeeping genes under specific conditions. A previous study reported that *SAND1* was the optimal reference gene for roots and leaves from plants of *Gentiana macrophylla* exposed to abiotic stress^47^. Similarly, we found that *SAND* also was the stable reference gene in different tissues of *Taxus* spp. Moreover, *RBCL* was the most suitable reference gene under ABA treatment, while *ARP2* also showed better performance among eight candidate reference genes under COR and MeJA treatments.

Taxol production by *Taxus* cells was induced upon incubation of the cell culture at the optimal SA concentration^5,48^. Our results indicated that the expression of *TcMYC* was down-regulated after SA treatment. This suggested that *TcMYC* played negative regulating role in the complex signaling network of SA-induced taxane biosynthesis. Moreover, evaluation of the expression of *TcMYC* under SA treatment (Fig. 4), definitely proved that gene expression results were more accurate with stable reference genes than with unstable reference genes.

Recently, high-throughput sequencing technology has been developed rapidly and has been already widely used in genomics and functional genomics. Thus, a great deal of genomes and transcriptome sequences and gene expression data have been generated by sequencing, that are helpful and effective to quickly screen ideal internal reference genes. With the genome and transcriptome sequences, some new reference genes have been explored and widely used with higher expression stabilities^49–53^. This can benefit to evaluate new and common candidate reference genes in *Taxus*. To examine the transcripts level of a few target genes, RNA-seq method costs are higher compared to qRT-PCR. Moreover, qRT-PCR is also usually used to verify the results of unigene expression of RNA-seq. In the present study, the sequences of novel reference genes, *RBCL*, *ARP2*, and *SAND*, were obtained from transcriptome data, and performed better than other traditional reference genes. If economy permits, using transcriptome data to obtain the most suitable reference genes is a feasible and time-saving strategy that not only provides sequences, but additionally, it seems highly convenient for screening good housekeeping genes.

In conclusion, this is the first study in which a set of candidate reference genes was analyzed in terms of their expression stability in *Taxus* spp. Although the final ranking of reference gene of five different statistical algorithms showed slightly different, however by combining and analyzing the data together, *GAPDH1* and *SAND* are most stable reference genes under all experimental conditions. This study also provides a directive foundation for further analyses of gene expression involved in the complex mechanisms of the taxol-biosynthesis pathway.

## Methods

### Plant materials and stress treatments

*Taxus chinensis* var. *mairei* plants were grown in soil pots for 5 years in the greenhouse of the Chinese Academy of Forestry. The tissue samples including roots, stems, leaves, phloems, and xylems were collected immediately frozen in liquid nitrogen, and then stored at − 80 °C for RNA extraction. These samples were collected from three different trees as biological replicates.

A *Taxus* × *media* cell line was selected as material to be induced by elicitors, which grown on B5 solid medium as described previously^54^. For inducing treatments, after filter-sterilized, the elicitor (ABA, COR, MeJA, SA, and ETH) was added to the medium before solidification, with the final concentration of these elicitors was 100 μM, 1 μM, 100 μM, 100 μM, and 30 μM, respectively^10,55–57^. Cells were harvested at 0, 2, 6, 12, 24, and 48 h after each treatment. All cell samples were also collected immediately frozen in liquid nitrogen and stored at − 80 °C. Cells in three culture flasks were collected as biological replicates.

### Total RNA extraction and cDNA synthesis

Total RNA was extracted from the samples of tissues and cells line samples with the Column Plant RNAout 2.0 kit (TIANDZ, Beijing, China), which was treated with DNase I to remove the genomic DNA. The total RNA quantity and the purity were assessed with NanoDrop 8000 spectrophotometer (Thermo Fisher SCIENTIFIC, Waltham, MA, USA) and electrophoresis on 1.5% agarose gels. Using the FastQuant RT kit with gDNase (TIANGEN, Beijing, China) cDNA synthesis was performed with 1 μg total RNA. For use as templates for RT-PCR and qRT-PCR, cDNA samples were diluted four-fold in sterile water and stored at − 20 °C.

### Candidates reference gene selection and primers design

There were 23 candidate reference genes were selected and designed primers with Primer premier 6 software for the qRT-PCR experiments, including 17 reference genes were obtained from our previous RNA-seq data (unpublished), and six reference genes previously had been used in studies of gene expression in *Taxus* spp. However, of these, there were eight reference genes were screened out, which produced the single predicted band and a unique absorption peak though RT-PCR and qRT-PCR reaction (Fig. 1, Table 1). Among the eight candidate reference genes, *ARP2* (encoding actin-related protein) (accession number MK281332), *GAPGH1* (accession number MK281329), *RBCL* (Ribulose-1,5-bisphospate carboxylase/oxygenase) (accession number MK281331), and *SAND* (Sand protein) (accession number MK281330), were selected from our previous transcriptomic data (unpublished), and the reference genes *Actin*, *TBC41*, *18S rRNA*, and *GAPDH2* previously had been used in studies of gene expression in *Taxus* spp.^25–28^, while they were not evaluated in different tissues and different treatments, such as ABA, COR, MeJA, SA, and ETH involved in this study. The specificity of primer pairs was verified by electrophoresis on 1.5% agarose gels and analysis of the dissociation (melt) curves. According to the standard curve method, the PCR amplification efficiencies and regression coefficients (*R*^2^) of each primer pair were calculated using a cDNA sample with serial four-fold serial dilution, which was reverse transcripted from the total RNA of *T*. × *media* cell.

The target gene *TcMYC* was selected to confirm the expression difference with stable and unstable reference genes under SA treatment. The primer pairs of *TcMYC* were synthesized according to our previous study^9^ (Table 1).

### RT-PCR and qRT-PCR assay

RT-PCR analyses were performed with a reaction volume of 25 μL containing: 12.5 µL 2 × Taq PCR Master Mix (TIANGEN, Beijing, China), 3 µL of cDNA, 1 μL of gene-specific primer pairs (10 μM), and 7.5 μL sterile ddH_2_O. The amplification condition was 3 min at 94 °C for initial denaturation; 30 cycles of 30 s at 94 °C, 30 s at 60 °C, and 1 min at 72 °C, and the final extension at 72 °C for 5 min, finally, take 6 μL products for 1.5% agarose gel electrophoresis detection.

Using a Roche Light Cycler 480 (Roche, Basel, Switzerland), qRT-PCR analyses were performed with a reaction volume of 10 μL containing: 5 µL SYBR FAST qRT-PCR Kit Master Mix (2×) (KAPA, Sigma-Aldrich, St. Louis, MI, USA), 1.2 µL of cDNA, 1.3 μL of gene-specific primer pairs (5 μM), and 2.5 μL sterile ddH_2_O. The condition of the reaction was 95 °C for 3 min, 45 cycles of 95 °C for 10 s, 60 °C for 20 s and 72 °C for 30 s to calculate Ct values. Three biological and technical replicates for the sample of each treatment and tissue were used in the experiments.

### Gene expression stability analysis

Among the test reference genes of each sample, the Ct values were analyzed with Delta Ct (ΔCt), BestKeeper (version 1.0; https://www.gene-quantification.de/bestkeeper.html), geNorm (version 3.4; http://medgen.ugent.be/~jvdesomp/genorm/), and NormFinder (version 0.953; https://moma.dk/normfinder-software) methods^16,31,58,59^. Moreover, comprehensive evaluation was also carried out with the Ct values using the RefFinder tool (http://leonxie.esy.es/RefFinder/?type=reference). Using the 2^−ΔΔCT^ method^12^ and one-way ANOVA (SAS 9.2 software), the *TcMYC* expression data and statistical analyses was analyzed.

## Supplementary Information

Supplementary information.

## Data Availability

All data generated or analyzed during this study are included in this published article and its “Supplementary Information” Files.
