# Supplementary material for: Selection and validation of reference genes for quantitative gene expression normalization in Taxus spp
Source: Sci Rep. 2020 Dec 17;10:22205. doi: 10.1038/s41598-020-79213-1 (PMC7747704; doi:10.1038/s41598-020-79213-1)
Supplement: Supplementary file 1 — Supplementary information. [file 41598_2020_79213_MOESM1_ESM.pdf]

Title

Selection and validation of reference genes for quantitative gene expression normalization in *Taxus* spp.

Authors

Kaikai Zhang[1,2], Wei Fan[3], Duanfen Chen[2], Luyuan Jiang[1,2], Yunfeng Li[4], Zhiwang Yao[2], Yanfang Yang[1*] & Deyou Qiu[1*]

Address

1. State Key Laboratory of Tree Genetics and Breeding, Key Laboratory of Tree Breeding and Cultivation of State Forestry Administration, The Research Institute of Forestry, Chinese Academy of Forestry, Beijing 100091, China
2. College of Horticulture, Agricultural University of Hebei, Baoding, 071001, China
3. State Key Laboratory of Tree Genetics and Breeding, Chinese Academy of Forestry, Beijing 100091, China
4. College of Agriculture, South China Agricultural University, Guangzhou, 510642, China.

[*] Correspondence: echoyyf@caf.ac.cn; qiudy@caf.ac.cn

Supplementary Figure

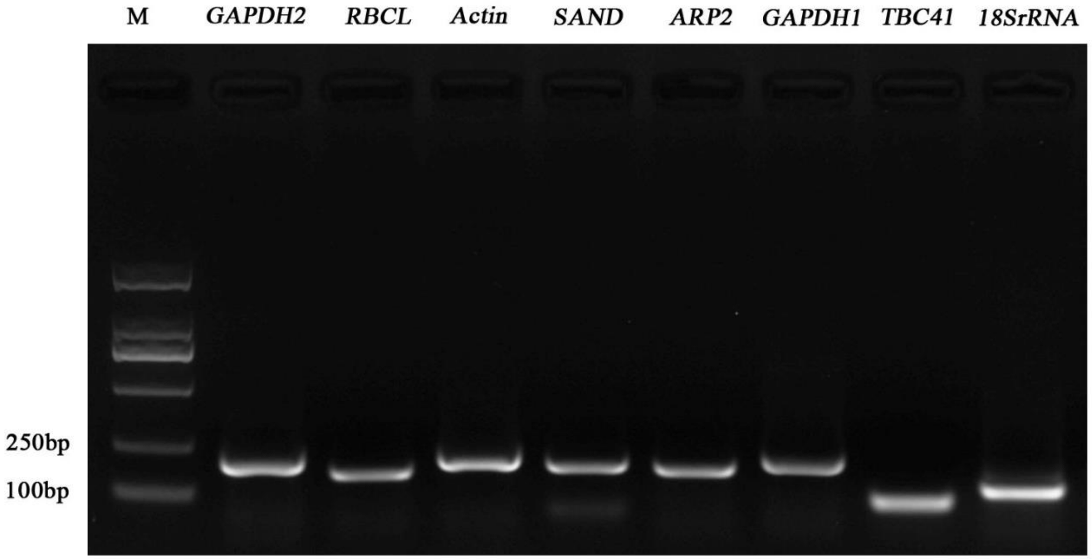

Supplementary Figure S1. Full-length gel of eight candidate reference genes PCR products. Lane M, DNA 2000 Marker. The figure was generated by ChemiDoc Touch Gel Imaging System (version 1708370; https://www.bio-rad.com/).

Supplementary Table

Supplementary Table S1. Expression stability and ranking of eight candidate reference genes by geNorm, NormFinder, BestKeeper and ΔCt in the different treatments

Treatments*	geNorm			NormFinder		BestKeeper		Delta Ct	
	Rank	Gene	Stability M	Gene	Stability value	Gene	StdDev	Gene	StdDev
ABA	1	*RCBL*	0.189	*GAPDH1*	0.025	*ARP2*	0.177	*RBCL*	0.399
	2	*GAPDH1*	0.189	*RBCL*	0.096	*18S rRNA*	0.195	*GAPDH1*	0.400
	3	*ARP2*	0.232	*18S rRNA*	0.114	*RBCL*	0.202	*18S rRNA*	0.437
	4	*18S rRNA*	0.254	*ARP2*	0.199	*GAPDH1*	0.213	*ARP2*	0.455
	5	*SAND*	0.280	*SAND*	0.210	*SAND*	0.236	*SAND*	0.468
	6	*Actin*	0.310	*Actin*	0.278	*Actin*	0.374	*Actin*	0.522
	7	*GAPDH2*	0.394	*GAPDH2*	0.340	*GAPDH2*	0.532	*GAPDH2*	0.634
	8	*TBC41*	0.533	*TBC41*	0.629	*TBC41*	0.792	*TBC41*	0.951
COR	1	*ARP2*	0.298	*18S rRNA*	0.146	*RBCL*	0.298	*18S rRNA*	0.461

	2	*SAND*	0.298	*ARP2*	0.173	*18S rRNA*	0.328	*ARP2*	0.470
	3	*RBCL*	0.334	*GAPDH2*	0.205	*ARP2*	0.361	*SAND*	0.506
	4	*18S rRNA*	0.379	*GAPDH1*	0.231	*SAND*	0.407	*GAPDH2*	0.517
	5	*Actin*	0.408	*Actin*	0.234	*GAPDH1*	0.474	*Actin*	0.519
	6	*GAPDH2*	0.429	*SAND*	0.239	*Actin*	0.563	*GAPDH1*	0.520
	7	*GAPDH1*	0.445	*RBCL*	0.290	*GAPDH2*	0.579	*RBCL*	0.550
	8	*TBC41*	0.548	*TBC41*	0.558	*TBC41*	0.769	*TBC41*	0.853
MeJA	1	*GAPDH1*	0.198	*GAPDH1*	0.097	*18S rRNA*	0.102	*GAPDH1*	0.397
	2	*ARP2*	0.198	*SAND*	0.130	*ARP2*	0.194	*ARP2*	0.401
	3	*SAND*	0.213	*RBCL*	0.131	*GAPDH1*	0.226	*SAND*	0.401
	4	*18S rRNA*	0.245	*ARP2*	0.138	*SAND*	0.255	*18S rRNA*	0.427
	5	*RBCL*	0.272	*18S rRNA*	0.150	*RBCL*	0.308	*RBCL*	0.436
	6	*Actin*	0.317	*GAPDH2*	0.213	*Actin*	0.405	*GAPDH2*	0.511

	7	*GAPDH2*	0.360	*Actin*	0.324	*GAPDH2*	0.442	*Actin*	0.547
	8	*TBC41*	0.510	*TBC41*	0.646	*TBC41*	0.849	*TBC41*	0.961
SA	1	*18S rRNA*	0.180	*SAND*	0.081	*18S rRNA*	0.134	*SAND*	0.422
	2	*GAPDH1*	0.180	*GAPDH1*	0.117	*GAPDH1*	0.167	*GAPDH1*	0.429
	3	*SAND*	0.236	*ARP2*	0.167	*SAND*	0.227	*ARP2*	0.463
	4	*ARP2*	0.264	*18S rRNA*	0.183	*ARP2*	0.293	*18S rRNA*	0.476
	5	*RBCL*	0.339	*RBCL*	0.256	*RBCL*	0.343	*RBCL*	0.542
	6	*GAPDH2*	0.408	*GAPDH2*	0.319	*Actin*	0.475	*GAPDH2*	0.602
	7	*TBC41*	0.471	*TBC41*	0.407	*TBC41*	0.491	*TBC41*	0.687
	8	*Actin*	0.552	*Actin*	0.500	*GAPDH2*	0.522	*Actin*	0.793
ETH	1	*GAPDH1*	0.262	*Actin*	0.129	*RBCL*	0.174	*Actin*	0.399
	2	*Actin*	0.262	*18S rRNA*	0.139	*SAND*	0.189	*18S rRNA*	0.415
	3	*18S rRNA*	0.271	*GAPDH1*	0.174	*Actin*	0.288	*GAPDH1*	0.424

	4	*ARP2*	0.296	*ARP2*	0.209	*18S rRNA*	0.298	*ARP2*	0.455
	5	*SAND*	0.358	*SAND*	0.239	*GAPDH1*	0.365	*SAND*	0.472
	6	*RBCL*	0.393	*GAPDH2*	0.252	*ARP2*	0.434	*GAPDH2*	0.506
	7	*GAPDH2*	0.425	*RBCL*	0.315	*GAPDH2*	0.520	*RBCL*	0.547
	8	*TBC41*	0.486	*TBC41*	0.416	*TBC41*	0.594	*TBC41*	0.672
Tissue	1	*18S rRNA*	0.346	*18S rRNA*	0.052	*18S rRNA*	0.473	*SAND*	0.867
	2	*SAND*	0.346	*SAND*	0.120	*SAND*	0.508	*18S rRNA*	0.904
	3	*ARP2*	0.495	*ARP2*	0.375	*GAPDH2*	0.570	*ARP2*	0.986
	4	*TBC41*	0.635	*Actin*	0.585	*ARP2*	0.659	*GAPDH2*	1.082
	5	*GAPDH1*	0.728	*TBC41*	0.629	*Actin*	0.729	*TBC41*	1.161
	6	*Actin*	0.900	*GAPDH2*	0.648	*TBC41*	0.839	*Actin*	1.190
	7	*GAPDH2*	0.986	*GAPDH1*	0.756	*GAPDH1*	1.056	*GAPDH1*	1.275
	8	*RBCL*	1.219	*RBCL*	1.246	*RBCL*	1.109	*RBCL*	1.904

Note: ABA, abscisic acid; COR, coronatine; MeJA, methyl jasmonate; SA, salicylic acid; ETH, ethylene. *SAND*, Sand protein; *ARP2*, Actin-related protein; *RBCL*, Ribulose-1,5-bisphospate carboxylase/oxygenase; *GAPDH1/2*, Glyceraldehyde-3-phosphate dehydrogenase; 1*8S rRNA*, 18S ribosomal DNA; *TBC41*, 3,5-epimerase-4-reductase.
